# A qualitative investigation of health benefits through a modified Taekwondo activity among nursing home residents

**DOI:** 10.1186/s12877-023-03749-w

**Published:** 2023-04-17

**Authors:** Junhyoung Kim, Yongseop Kim, Dong-Chul Seo, Sua Han

**Affiliations:** 1 School of Public Health, Indiana University, Bloomington, IN 47405 USA; 2 Department of Sports Coaching, Osan University, Osan, South Korea

**Keywords:** Taekwondo intervention, Physical health, Cognitive ability, Social health, Mental health, Nursing home resident

## Abstract

**Background:**

A few studies suggest that Taekwondo is an effective intervention in increasing physical functions among older adults. This study is intended to focus on a multitude of health benefits of participation in a modified Taekwondo activity for nursing home residents in the U.S.

**Methods:**

This qualitative study used semi-structured, in-depth interviews with seven participants consisting of 2 males and 5 females older adults from a community nursing home. The interview protocol included content mapping and content mining interview questions. The study followed the five steps of constant comparative analysis.

**Results:**

Four main themes were identified as health benefits resulting from a modified Taekwondo participation: (a) promoting mental health, (b) increasing physical functions, (c) stimulating cognitive abilities, and (d) facilitating positive social interaction.

**Discussion:**

This study indicates modified Taekwondo can be instrumental in promoting their physical functioning, cognitive functioning, social interactions, and mental health. Practical implications and further discussion are addressed in this paper.

Being physically active is considered the most important goal that health researchers have established for nursing home residents. A growing body of literature supports this goal, showing that participation in leisure-time physical activity increases physical functions, mental health, and cognitive abilities and provides social benefits, all of which contribute to successful aging among nursing home residents [1–4]. In spite of these benefits, however, physical inactivity is prevalent among nursing home residents, who constitute the population at highest risk for critical health-related problems associated with physical inactivity [5–7]. Moreover, physical inactivity has a direct influence on loss of lower body muscle mass, which increases the risk of falling for the elderly over the age of 70 [8, 9]. Much empirical research has demonstrated the role of physical activity in both improving mental and physical health and decreasing the frequency of falls of nursing home residents [4, 10, 11].

To reduce health and safety issues related to physical inactivity, Taekwondo, a form of Korean traditional martial arts, can be an effective means of engaging nursing home residents in physical activity. According to Kim [12], Taekwondo has historically promoted the interconnectedness of physical and psychological fitness in Eastern countries, and it is practiced worldwide today. Previous researchers have found that Taekwondo strengthened the synthesis of the mind and body among adolescents with and without disabilities by cultivating discipline, self-control, and self-reflection in association with developing physical strength and body control, including balance and coordination [13–15]. These studies suggest that Taekwondo may also be an effective intervention for promoting physical and mental health among older participants.

To explore the possible health benefits of Taekwondo participation for older adults living in nursing homes, further research is needed. As indicated, nursing home residents are strongly encouraged to participate in physical activity for their mental health and successful aging. While healthcare professionals design and implement a variety of exercise and recreational programs for nursing home residents, however, Taekwondo may be unfamiliar in these settings in the U.S., and therefore its effects on the residents’ health and wellbeing are understudied. Also, among the important features of Taekwondo are meditation practices rooted in Korean culture and language, which may also provide mental health benefits that have not been investigated in previous studies. Therefore, this study used a qualitative research methodology to examine the variables that affect residents' social interaction, mental health, cognitive ability, and physical activity in nursing homes. Additionally, we gathered suggestions and ideas to aid in the creation of specialized intervention programs meant to boost physical activity and lower sedentary habits in nursing home residents.

## Possible health benefits of Taekwondo for nursing home residents

Previous studies have found that participating in a Taekwondo program can promote mental health and life satisfaction [16–18]. Iso Aloha and Park [16] reported that Taekwondo learners experienced social support and reduction of the adverse effects of stress on their physical and mental health. Weiss [18] explored the relationships between participants’ Taekwondo experience and their body image perceptions, self-esteem, and overall mental health and concluded that Taekwondo is positively associated with self-esteem, psychological motivations, and affirmative body image. According to Kim, Heo, and Dattilo [17], individuals who were more engaged in Taekwondo activity reported higher life satisfaction and more positive health perception than those who were less engaged.

In addition, Kim et al. [13] found that after participating in an 8-week Taekwondo training program, 14 children with autism spectrum disorder exhibited significantly greater improvement in one-leg stance balance with their eyes closed than did nonparticipants. Other researchers have found that adolescents who participated in Taekwondo intervention significantly increased self-regulation skills and mental health while reducing self-destructive behaviors [14]. Petrovic [19] found that Taekwondo practice helped undergraduate students to reduce stress and expand their sense of courage, self-respect, and determination to improve themselves and their lives.

Given the strong evidence in the literature that Taekwondo can be beneficial for health and wellbeing, it is proposed that these benefits can be extended to nursing home residents. Compared to other traditional mind–body practices in Eastern cultures such as Tai Chi, Yoga, and breathing techniques, the unique characteristic of Taekwondo is that it includes vigorous but low-impact exercise as a component, such various stances and punching, blocking, and kicking moves. A few studies have demonstrated the effectiveness of Taekwondo as an intervention program for older adults. For instance, Cho and Roh [20] found that a Taekwondo intervention improved older Korean women’s physical fitness and cognitive function after 12 weeks. Similarly, Cromwell and his colleagues [21] found that older adults who participated in Taekwondo showed significantly greater improvements in walking velocity and multidirectional reaching abilities than a comparison group who did not practice Taekwondo. Furthermore, Youm et al. [22] found that Taekwondo participation was instrumental in enhancing the static balance control of 30 female older adults.

To extend the existing literature on the benefits of Taekwondo for older adults, this study focused specifically on the health-related benefits of participation in Taekwondo activity for nursing home residents in the U.S. Considering the characteristics of geriatric population such as mobility issues and debilitating physical functions, our research team modified the intensity, length, and series of Taekwondo movements (e.g., sitting stances in Taekwondo). To gain a deeper understanding of modified Taekwondo experiences of this population, a qualitative methodology was employed to capture their perspectives and their voices. The goal of this qualitative investigation was to provide researchers with preliminary information on the health benefits of modified Taekwondo for nursing home residents as well as their challenges and adverse events during the participation as a basis on which to design a follow-up experimental study to further assess health outcomes through modified Taekwondo.

## Methodology

This qualitative study employed semi-structured, in-depth interviews to explore the health benefits experienced by nursing home residents as a result of modified Taekwondo participation. This method allowed us to explore the personal and social experiences and capture the unique perspectives of nursing home residents in relation to their modified Taekwondo participation [23].

## Participants and data collection

The research team contacted the director of the target nursing home and obtained permission to place flyers containing a brief description of the study on their notice boards. Interested parties contacted the research team by email or phone. At an orientation presentation, the research team provided prospective participants with consent forms and information (e.g., the study’s purpose, activities, and withdrawal procedures). Our research team obtained informed consent from all participants before their participation. The research team had developed an 8-week modified Taekwondo program, which was implemented with seven participants by a certified Taekwondo instructor with volunteers who provided extra assistance. After completion of the program, the participants were asked to share their modified Taekwondo experiences in semi-structured interviews.

This study utilized a criterion sampling strategy [24]. The inclusion criteria of the study participants are who (a) were residing in a nursing home, (b) had no prior Taekwondo experience, and (c) had no medical record of serious mental illnesses (such as Alzheimer’s disease and related dementia). Before their participation, we conducted preliminary interviews to verify each participant’s cognitive ability to communicate. None of the potential participants demonstrated communication or cognitive challenges. Among 9 prospective participants, seven, who ranged in age from 76 to 84, participated in this study. This sample size was determined sufficient on the basis of data saturation when no new themes emerged after analysis of the six interviews, as suggested by Guest et al. [25] The average age was 79.6; five were female; four were married, two were widowed, and one was single. All of them had achieved at least a baccalaureate degree. Additional demographic informationg is provided in Table 1. In this study, pseudonyms were used to identify participants. The University Institutional Review Board approved these procedures (#1,910,509,624).

**Table 1:** Demographic information

No	Name	Gender	Age	Marital Status	Education Level	Perceived Health
1	Brandon	Male	78	Married	College graduate	Good
2	Ryan	Male	77	Married	Graduate school	Very good
3	Mary	Female	76	Married	Graduate school	Very good
4	Rosemary	Female	84	Widowed	Graduate school	Excellent
5	Stella (Susanne)	Female	76	Married	College graduate	Good
6	Sandy	Female	82	Single	Graduate school	Good
7	Sonia	Female	84	Widowed	College graduate	Excellent

## Adapted Taekwondo intervention

The adapted Taekwondo intervention is aimed at improving the overall health of residents living in nursing homes. The intervention group participated in a 50-min Taekwondo exercise class two times a week for eight weeks in the community nursing home. The intensity of the program was gradually increased over time to help participants achieve success. The intervention was led by certified Taekwondo instructors from Indiana University. Each class started with a 5-min warm-up, followed by 15 min basic Taekwondo movements such as punching, blocking, and a variety of stances, 15 min of a form Taekwondo Taeguek 1 jang, which consist of series of basic movement form, and ending with 10 min of flexibility/ stretching cool-down, and meditation. The warmup activity consisted of light walking, joint rotation (neck, shoulder, hip, knee, and ankle), arm and leg stretching, and other related exercises. The basic Taekwondo movements included middle, high, and low punching, and blocking. A chair was used to provide a safe support when needed. Also, 4 volunteers stood aside by participants to assist participants when they did stances and series of movements. The program was delivered as planned and rehearsed jointly by instructors and all volunteers.

## Interview protocol

The research team developed the interview protocol based on discussions with a professional Taekwondo consultant and a recreational therapist who were experienced in working with nursing homes. Due to COVID-19, interviews were conducted by telephone. The interviews lasted between 30–60 min. The interview protocol included content mapping and content mining interview questions. Examples of content mapping questions were “Could you tell me about your overall experience in learning Taekwondo?” “What benefits did you experience when participating in Taekwondo program?” and “What challenges did you experience when participating in Taekwondo program?” These questions allowed the investigators to form a general overview of participants’ experiences and the benefits and challenges they encountered. Content mining questions were used to explore specific health benefits from Taekwondo participation. They included “Based on your experiences, what role, if any, has this activity had in helping you deal with challenges in your life?” “Based on your experiences, what role, if any, has this activity had in helping you deal with challenges in your life?” and “How does this activity contribute to your happiness?” After each interview, the participants completed a brief demographic questionnaire eliciting gender, age, and educational background.

## Data analysis and trustworthiness

The research team applied constant comparative analysis as proposed by Creswell [25] and earlier described by Taylor and Bogdan [26]:

In the constant comparative method the researcher simultaneously codes and analyses data in order to develop concepts; by continually comparing specific incidents in the data, the researcher refines these concepts, identifies their properties, explores their relationships to one another, and integrates them into a coherent explanatory model” (p. 126).

The five steps of constant comparative analysis suggested by Creswell [26] are (a) creating raw data, (b) preparing data analysis, (c) understanding and analyzing each data item, (d) generating general themes with direct quotes, and (e) interpreting the themes. We followed each step, compared and contrasted each data item with others concurrently to ensure confirmability, dependability, and transferability of the findings, and reached consensus on interpretation. 

To increase the trustworthiness of the data analysis, we conducted member-checking with participants, as suggested by Peterson, Sword, Charles, and DiCenso [27], to verify the accuracy of our interpretations. In phone meetings, we presented an overview of our findings and interpretations, and all the participants agreed with them. In addition, our research team had rigorous training and extensive experiences using qualitative methods.

## Findings

Based on the participants’ statements concerning their experiences in modified Taekwondo, four main themes were identified as health benefits resulting from modified Taekwondo participation: (a) promoting mental health, (b) increasing physical functions, (c) stimulating cognitive abilities, and (d) facilitating positive social interaction. These benefits can show evidence that modified Taekwondo can be used as a program for promoting these health benefits among nursing home residents.

## Promoting mental health

Mental health is the most salient theme that emerged from the data. All of the participants indicated that they experienced mental health benefits through modified Taekwondo participation especially in the form of enjoyment as well as in self-confidence, stress reduction, and cultural growth. Based on their personal statements and experiences, a sense of enjoyment was the large proportion of mental health. They mentioned the inherent characteristics of modified Taekwondo that involved a series of physical movements (e.g., kicking, punching, blocking) and meditating. These features facilitated their interests and enjoyment. For example, Mary (female, 76) said,

*This is something I never had any interest in before, but I did enjoy it and I think one of the things that made it quite enjoyable was the way your relentless and encouraging attitude. It really made it a fun time.*

She also indicated that she was willing to continue participating in a modified Taekwondo activity and encouraged other residents to join the program.

Another aspect of enjoyment is learning about a new culture and language, which is associated with modified Taekwondo. All of the participants stressed that learning about Eastern culture and language as elements of modified Taekwondo added to the pleasure they took in the practice. For example, Brandon (male, 78) stated that he enjoyed learning new ways of communicating such as bowing to show respect and how to count in Korean. In addition, participants shared their experience of shouting *Ki-Hap*, a “spiritual yell,” when they performed various techniques. For example, Stella (female, 76) stated,

*You know I think that [Ki-Hap] is psychological. I think that was pretty good. We were all kind of shouting out at the same moment. Whatever problems we were thinking about, . . . you know, we just kind of let it [all] go, so that felt pretty good, really. And we were all having a good time doing that.*

By shouting out with other participants, she not only enjoyed the moment but also felt that she received spiritual energy, which can be illustrated by the power within the spirit and the mind. In a similar manner, Sandy (female 82), who had been reluctant to participate in this activity because of its unfamiliarity, said that *Ki-Hap* helped her immerse herself in the experience. She shared that shouting loudly with other participants created powerful energy.

Most participants mentioned that participating in a modified Taekwondo activity reduced their stress and increased their positive feelings and confidence. Brandon (Male, 78) stated that “*meditation is a good way to get rid of my unpleasant thoughts*.” In addition, Sandy (female 82), said that as she progressed, she felt more confident and less stressed by nursing home life. Similarly, Ryan (male, 77) stated that he relieved his stress and gained confidence in the physical exercises of modified Taekwondo demonstrations (e.g., kicking, punching, and shouting *Ki-Hap*) through it. It appears that their engagement in modified Taekwondo made them feel accomplished and confident. As Mary (female, 76) said, “*Well, we were always happy when we left. We left in a really good, upbeat mood. It was a good experience. I would do it again and I would encourage others to do it*.”

Some participants compared modified Taekwondo with other mind–body activities and believed that it offered more health benefits, including mental health. For example, Stella (female, 76), who had participated in a Tai-Chi program, said that she preferred Taekwondo because it was more fun and invigorating, while Tai-chi was focused on slower movements. Similarly, Sandy (female 82), stated,

*Most of the Taekwondo, you see, is usually quite quick and energetic. At first, I didn’t know how that would translate for me. It has very different movements from yoga movement, which is so slow. I found that Taekwondo was very beneficial, and I really liked it. It made me feel very refreshed. I was very pleased with the way it turned out.*

As these excerpts illustrate that the participants felt they benefitted from the combination of educational, meditational, and physical aspects of modified Taekwondo, which supported their mental health through learning, reduction of stress, and stimulation through energetic movement.

## Increasing physical functions

All of participants mentioned that performing various movements such as kicking, punching, and stances helped increase physical balance. For example, Brandon (male, 78) stated that he enjoyed practicing modified Taekwondo for the exercise, which he found increased has balance and sense of physical presence. Jane (female, 74), who had recently undergone the joint surgery, stated,

*I think that Asian martial arts are definitely effective for recovering body balance after joint surgery. For example, basic Taekwondo postures are composed of various kicking and punching poses, which require a high level of balance. I actively encourage martial arts for rehabilitation of patients who have undergone joint surgery.*

In a similar manner, Stella (female, 76) stated, “*My left leg is pretty weak, and my right leg is pretty strong, and I felt that Taekwondo helped me adjust my balance between the two sides*.” After improving her balance through modified Taekwondo, she believed that her gait had become more stable. Along with improved physical balance, some of the participants also indicated that engaging in modified Taekwondo had improved their eye-hand coordination.

Participants also said that modified Taekwondo helped them become more physically active. To improve their modified Taekwondo techniques and skills, they invested their time and energy into practicing modified Taekwondo basic skills they learned and. By practicing modified Taekwondo, they believed that they became physically active. Determined to advance his skills and techniques through additional practice and meditating on what he learned from modified Taekwondo participation, Brandon (male, 78) also engaged in self-learning by watching video clips that demonstrated modified Taekwondo movements and techniques. Also, Mary (female, 84) also described her efforts to master the precise movements of the art, finding some more challenging than others but persisting nevertheless:

*After the very first time I noticed a couple muscles I hadn’t used for a while, but I didn’t find it difficult to do. It was just difficult to do precisely and knowing when to do what. What came after what. I never got the kicks with the tapping the foot flat. I don’t think I figured that one out. But I know what I’m supposed to do, it’s just carrying it out that’s the issue. That takes practice.”*

Sandy (female, 82) stated, “*I think I felt my muscles became more flexible and it seemed to be a good feeling, moving like that*.” Also commenting on the holistic effects of modified Taekwondo, Sonia (female, 84) said, “*I think it was probably everywhere. I felt really good everywhere. With what we did we moved arms and legs, and everything. We were using our whole, bodies and I think it was extremely beneficial to do that*.”

These examples show that the participants felt that they were more physically active and experienced improvements in physical strength and balance. From these testimonies, it may be inferred that modified Taekwondo can be an effective therapeutic program to increase their physical function.

## Stimulating cognitive abilities

Stimulating cognitive abilities is another theme that emerged from participants also reported that following a series of physical movements helped to stimulate their cognitive functioning. To acquire the physical movements of modified Taekwondo, they had to memorize the patterns of arm, leg, and foot movements and practice them to achieve mastery. For example, Brandon (male, 78) stated,

*I think probably it stretched me in terms of trying to use my memory, particularly in terms of doing exercise patterns. That was an interesting experience. It took me a while sort of internalize [the movements] into memory and then to translate that memory back into physical activity. But it worked.*

In addition, Jane (female, 74) said that learning a series of movements helped her memory skills, stating “it helps me remember what goes before and what comes after. The whole sequence was fun to learn.” With practice, she became more familiar with the sequencing of modified Taekwondo movements. Ryan also found that following the sequences of modified Taekwondo helped improve his memory skills.

With regard to the cultural aspects, all of the participants were interested in learning Korean words through the modified Taekwondo activity, particularly counting in Korean, which helped them remember the order of movements and follow the sequence. Sandy (female, 82) stated that she found learning the basic Korean vocabulary for the positions and movements was cognitively stimulating. Ryan (male, 77) also developed an interest in the language of modified Taekwondo:

*It was interesting learning some of the language and terms. When I was looking at some of the videos, they were all very similar using the same terms and teaching the same forms. There are a couple videos where they go much more into the language as far as all the different terms.*

He also considered his acquisition of the basic terms as cognitively beneficial.

The participants who embraced the challenges associated with modified Taekwondo concentrated on learning and practicing the techniques and skills. They believed that the effort they invested in improving their modified Taekwondo performance enhanced their mental functioning. For example, Mary (female, 84) stated that her ability to concentrate was enhanced by the challenge of learning Taekwondo techniques. Also, Jane (female, 74) stated,

*Learning Taekwondo was an interesting and pleasant challenge. I think I could see I was getting closer to learning it with each movement, but it was still a challenge for both the body and mind. I liked that… I am better at concentrating because of it.*

These statements reflecting their experiences with modified Taekwondo show that the participants were mentally stimulated by learning the movements of modified Taekwondo and related terms in Korean, following the sequence, and mastering the techniques, which required attention, memory, and concentration. They found these mental exercises beneficial for their cognitive functioning.

## Facilitating positive social interactions

All of the participants mentioned that engaging in modified Taekwondo as a group activity facilitated positive social interactions with other participants, the modified Taekwondo instructor, and volunteers. They reported that they valued sharing the experience of learning modified Taekwondo with others, developing friendships, and being encouraged by others to become more involved in the activity. For example, Brandon (male, 78) mentioned that modified Taekwondo encouraged his socialization with fellow participants through both their shared physical activities and the conversations they enjoyed about their experiences. Stella (female, 76) drew motivation from the group experience:

*I think this activity is most effective as a group activity. We were in a group, which I think gave us more energy. Being together created a situation in which all the people were having fun communicating and laughing, so that I kind of liked that.*

Some of participants valued their friendships with the modified Taekwondo instructor and volunteers, from whom they experienced receiving emotional and social support. Mary (female, 76) mentioned that she formed a sense of friendship, observing that she “*would have to say that the interactions with you [the instructor and volunteers] and the other students*” were her favorite part of the activity. Sonia (female, 84) also mentioned that the enthusiasm and support from the instructor motivated her to engage in and enjoy modified Taekwondo. After sessions, she gave him feedback about her modified Taekwondo experiences and shared some of her personal life experiences with him.

In a similar way, Jane (female, 74) mentioned that she appreciated the support she received from the instructor and volunteers, with whom she formed a sense of friendship. She mentioned that her grandson-in-law was associated with Korean culture, which heightened her interest in learning Taekwondo. Similarly, Brandon (male, 78) told the instructor, “*We really enjoyed you and your associates. They were very kind, very thoughtful and very helpful*.”

Based on examples and statements from participants, it can be inferred that the group format of the modified Taekwondo program allowed them to interact with others in a meaningful manner.

## Discussion

This qualitative study is the first to shed light on the health-related benefits associated with modified Taekwondo participation by nursing home residents. The results indicate that modified Taekwondo can be instrumental in promoting their physical functioning, cognitive functioning, social interactions, and mental health. In addition, participants reported that learning about Korean culture and language in association with modified Taekwondo stimulated their interest and gave them new cultural knowledge and understanding. These benefits can provide preliminary evidence to researchers that modified Taekwondo can be used as an intervention to physical, cognitive, and mental health benefits among nursing home residents.

Previous studies have provided evidence that physical inactivity among nursing home residents is associated with psychological challenges such as feelings of isolation, loneliness, and depression [5–7]. The findings of this study indicate that participants experienced reduced stress, greater confidence, and an increased sense of happiness as well as gained new cultural understanding, which stimulated them cognitively. These subjective benefits suggest that modified Taekwondo can be an effective program to ameliorate negative psychological states such as stress, and loneliness by experiencing sense of belonging and gaining self-control among nursing home residents. Thus, this study extends the findings of prior studies showing that adolescents who participated in modified Taekwondo interventions exhibited improved self-control and increased enjoyment to nursing home residents [13–15]. Participation in modified Taekwondo can increase their confidence, enjoyment, and experience of physical improvement, and in this way, it may positively affect the overall mental health of nursing home residents.

Prior studies exploring the benefits of Taekwondo activity such as personal growth, cultural understanding, multicultural appreciation [28, 29] have shown that participants experienced a reduction of negative stereotypes and became more culturally sensitive and resulting personal growth. The findings of this study support these benefits of the modified Taekwondo activity among nursing home participants, who were motivated to learn the movements because of the cultural elements embedded in the martial art practices.

The finding of this study that practicing modified Taekwondo can improve physical balance confirms prior research that has documented the role of physical activity in decreasing the frequency of falls of nursing home residents [4, 10, 11]. In particular, Kim et al. [13] implemented an 8-week Taekwondo program for 14 children with autism spectrum disorder, after which participants reported greater improvements in a single-leg stance balance with their eyes closed than did nonparticipants. Also, Sherrington et al. [11] suggested that one-third individuals aged over 65 years old are experiencing falling once or more annually, and it can be prevented by group-based exercise with 2 h a week for a 6-month period program. Chen [30] emphasized that age-adapted Taekwondo training is suitable for improving balance in older adults and reducing the fear of falling, and their training included a variety of basic movements that were performed progressively within participants’ abilities. The current study supports Kim et al. [13] and Sherrington et al. [11]’s results by demonstrating the positive effects of modified Taekwondo on physical balance among older adults.

Research has provided ample evidence that participation in leisure-time physical activities is effective in improving the cognitive functions of older adults [31, 32]. For example, Parisi et al. [33] found that recreational programs helped increase the cognitive capacity and cognitive performance of nursing home residents. Dodge et al. [34] also found that older adults gained cognitive benefits through leisure activities. This study demonstrates that following directions for physical movements of limbs and practicing the basic skills and techniques of modified Taekwondo can stimulate cognitive functions and cognitive capacity among nursing home residents.

As shown in prior studies, one of the important health benefits of group physical activity is that it allows older adults to experience a sense of belonging to a community and increases their positive social connections with others [1, 35, 36]. The current study confirms these findings by providing evidence that nursing home residents can have positive social interactions with other participants, the program instructor, and volunteers, which may increase their motivations to continue to engage in activity programs. This study also suggests that social benefits can enhance mental health by providing opportunities for enjoyment and happiness.

Some limitations of this study need to be addressed. First, this qualitative study designed to capture health benefits of a modified Taekwondo program among nursing home residents has the methodological limitations of involving only highly educated and predominantly Caucasian samples of nursing home residents, which curtails generalization of the results. Further experimentally designed studies with ethnically diverse samples are needed to ensure various perspectives on health outcomes of the intervention. Also, a larger sample size with a randomized controlled trial design is required to determine the efficacy of the clinical intervention. In addition, in this study the levels of each participant’s physical, emotional, and cognitive functions in relation to modified Taekwondo were not distinguished. Different effects on these functions may be associated with modified Taekwondo interests and motivations. Future researchers might examine how these variables are associated with the health benefits of such programs.

The Taekwondo instructor conducted the interviews with the participants, which may have resulted in a bias toward positive health outcomes in the study. To avoid researcher bias in evaluating participant experiences, our research team adhered to a rigorous process for data collection and data analysis. In addition, the present study focused on health benefits resulting from modified Taekwondo participation in nursing home residents. Considering that Taekwondo is based on a mind–body physical activity, future studies should evaluate the intensity of the activity and address health-related issues such as mobility and fatigue during the implementation.

In spite of these limitations, this study provides qualitative evidence that modified Taekwondo can be instrumental in promoting health benefits among nursing home residents. As indicated, the nursing home residents experience higher prevalence of physical inactivity and of psychological problems and concerns than older adults who are living independently in their communities [37–39]. The results of this study indicate that modified Taekwondo can be used as an intervention to increase the physical activity participation and ameliorate negative psychological consequences associated with confinement to nursing homes. In particular, modified Taekwondo is significantly associated with Korean cultural components that provide intellectual stimulation that can motivate participation. Overall, this study suggests that providing the invigorating bodily engagement in modified Taekwondo in a group format makes it a vehicle for improving physical, social, emotional, and cognitive functioning among nursing home residents.

## Data Availability

Because the participant permission form indicated that only the research team would have access to the data, the datasets collected and analyzed during this study are not publicly available, although analytic code lists are available from the corresponding author on reasonable request.
